# A Multifaceted Assessment of the Consultation Liaison Psychiatry Service Within a Regional Australian Hospital

**DOI:** 10.3390/healthcare12222250

**Published:** 2024-11-11

**Authors:** Clement Tan, Sandeep Reelh, Rahul Suri, Matthew Hiskens, Akshaya Ajit, Alok Rana

**Affiliations:** 1 Department of Psychiatry, Mackay Base Hospital, Mackay, QLD 4740, Australia; 2 College of Medicine and Dentistry, James Cook University, Mackay, QLD 4740, Australia; 3 Mackay Institute of Research and Innovation, Mackay Base Hospital, Mackay, QLD 4740, Australia

**Keywords:** consultation–liaison psychiatry, hospital psychiatry, service delivery, regional health

## Abstract

Background: Medical and mental health conditions have a reciprocal relationship, with each impacting the other. Consultation–liaison psychiatry (CLP) is a sub-speciality that addresses psychiatric comorbidities in the general hospital system and positively impacts hospital resources through its service aspects of ‘consultation’ and ‘liaison’. This multi-faceted evaluation aims to describe and evaluate the characteristics of a regional CLP service. Methods: Retrospective evaluation of all referrals accepted by CLP between May 2021 and 2022 were reviewed through the hospital’s electronic records. An evaluation tool was designed to assess multiple aspects of care, including the source of referrals, the reasons for referral, patient demographics, how quickly the patient was seen, information on their mental health, and the details and timeframe of CLP involvement. Findings: There were 147 patients included in this study. Doctors were the primary referrers (92%). A total of 94% of patients were reviewed within 24 h of the referral being received. Referral reasons were balanced across diagnostic clarification/review, medication review, and risk review. A large proportion of referrals were aged > 60 years. There was a large proportion of patients who lived rurally, requiring hospital transfer. CLP involvement was primarily required to provide a diagnosis (91% of patients) and provide pharmacological management advice (88%). Conclusions: The CLP service currently operates with limited resources, and without additional support, the sustainability of the service will become increasingly challenged due to population ageing. It is essential that we address this issue to ensure that the community’s needs are met effectively.

## 1. Introduction

The relationship between medical and mental health conditions is bidirectional, with each condition affecting the other. This co-existence of medical and psychiatric comorbidity can affect patients and the healthcare system negatively in many ways, such as higher resource usage, longer admission periods, increased representations to hospitals, and higher resource usage [1,2,3,4,5]. The recent international literature suggests that approximately 33% to 43% of patients admitted to medical wards have psychiatric illnesses that fulfil criteria set out by the DSM-V, and data from Australia indicate this rate is between 30% to 50% of patients [6,7]. As such, there is an urgent need for an effective and well-resourced team to manage these complex patients.

One strategy to manage patients with medical and psychiatric comorbidities is the provision of a consultation–liaison psychiatry (CLP) service [6,8]. CLP is a subspecialty of psychiatry that specialises in assessing, diagnosing, and managing the psychiatric comorbidities of patients hospitalised for medical conditions [9]. CLP services provide a positive impact on the health system through more comprehensive care, reduced length of stay (LOS), and improved cost efficiency [10,11]. In understanding the functioning of a CLP service in Australia and elsewhere, it is essential to delineate the terms ‘consultation’ and ‘liaison’. The ‘consultation’ aspect involves the assessment of inpatients referred within the general hospital system. This process is characterised by a timely bedside assessment, leading to the formulation of an impression/diagnosis and the tailoring of an individualised management plan. Subsequently, these management plans are communicated to the referring team, with follow-up reviews conducted as required [4,12]. Essentially, this aspect of the CLP service entails delivering mental health consultations and management to patients whose psychiatric conditions, though significant, may not be the primary reason for their hospital admission [4]. The ‘liaison’ aspect, on the other hand, centres on establishing professional hospital relationships with other hospital departments and professions. This involves the provision of education to equip hospital staff members with the necessary skills for managing general hospital inpatients with psychiatric comorbidities. Additionally, ‘liaison’ activities encompass attending unit meetings, participating in clinical case reviews, and engaging in discussions [12]. Collectively, within Australia, these efforts translate into improved mental health acumen, awareness, skills, and safety, ultimately providing support for inpatients with concomitant mental health issues and thereby improving overall patient outcomes [9].

There is an imperative need for quality improvement in healthcare services, as the majority of medical errors and inefficiencies are a result of faulty systems and processes rather than individual actions [13]. The Institute of Medicine has outlined six fundamental aims of healthcare services: effectiveness, safety, patient-centeredness, timeliness, efficiency, and equitability [14]. The primary objectives of assessing healthcare services include evaluating the impact of a service, whether it yields positive or negative effects, and gauging its adherence to evidence-based standards [15]. However, the measuring of service quality should be performed with available benchmarks to objectively identify true service performance and to identify improvements that are necessary for further service development [16]. Currently, Australia lacks national standards or benchmarks for CLP services, with the exception of staffing parameters [17].

Despite the evidence supporting CLP involvement with inpatients with psychiatric comorbidities, it has been shown in the international literature that CLP referral rates are much lower than required [6]. However, there is limited investigation of CLP referral and intervention rates in Australia. Therefore, the aim of this study was to evaluate the referral characteristics of a local CLP service using an evaluation tool that could potentially be reproducible in similar-sized services. To our knowledge, this is the first evaluation of its kind to be carried out for a CLP service within a regional Australian city.

## 2. Materials and Methods

### 2.1. Setting

The Mackay Base Hospital (MBH) is a public regional hospital with 251 beds, with a catchment area covering 90,364 km^2^, and servicing more than 170,000 people [18]. The CLP service at MBH was established in 2016 and operates with a single consultant, an advanced trainee of CLP, and a clinical nurse consultant. The CLP service operates on weekdays from the hours of 8am to 4pm. After-hours support is provided by the mental health on-call team, which is a consultant and a registrar. The team provides its services to the general hospital system, including departments such as obstetrics and gynaecology, paediatrics, emergency short stay, general surgery, general medicine, orthopaedics, intensive care, and coronary care. The CLP team handles new and existing referrals for routine or urgent mental health assessments, including conducting follow-ups as necessary. The CLP team provides its services for new or existing diagnoses of psychiatric conditions, including functional disorders, psychotic disorders, mood disorders, neuroses, cognitive disorders, or other psychiatric disorders associated with or resulting from a medical condition. This encompasses suicide and risk assessments, and the team provides advice on the initiation or adjustment of psychopharmaceutical drugs. In addition to its ‘consultation’ services, the CLP team also provides ‘liaison’ services, including providing psychoeducation with various hospital departments through either formal or informal teaching. Since its inception, the referrals received by the service have increased exponentially, which may reflect the recognised increased prevalence of mental health conditions associated with medical comorbidities [1,2,8,19]. However, the resourcing and staffing received by the CLP team have seen minimal changes.

### 2.2. Study Design

Ethical approval was provided by the Central Queensland Hospital and Health Service Human Research Ethics Committee (EX/2022/QCQ/88071). This retrospective evaluation involved a convenience sample of all CLP consultations spanning a one-year period from May 2021 to May 2022, and as descriptive but not inferential statistics were used, no a priori sample size calculation was required. The retrospective design allowed the evaluation of the service with regard to referrals and patient characteristics. The inclusion criteria encompassed all new referrals/consultations that were appropriately referred, and cases involving existing or recurrent consultations. The exclusion criteria comprised referrals that were duplicated and instances where patients legally discharged themselves against medical advice before the CLP review.

To ensure a comprehensive and impartial service evaluation, the evaluation tool was designed in several stages. Firstly, internal consultations were conducted with key health service stakeholders involved in service planning and resource allocations. Secondly, external consultations occurred with organisations and CLP services of comparable scale. Finally, the tool was trialled under the guidance of the CLP consultant and senior mental health clinicians. The tool is provided in Table 1.

### 2.3. Data Analysis

All data were entered into a Microsoft Excel sheet. Descriptive statistical data involving counts and percentages were calculated using Microsoft Excel.

## 3. Results

Of the 159 referrals directed to the CLP service during the study period, 147 met the inclusion criteria to be assessed through the evaluation tool. Of the twelve excluded referrals, five were due to referral duplication, three were due to the patients discharging against medical advice, and four were made in error on the iEMR system. The results section is delineated into the three sections of the evaluation tool.

### 3.1. Referral

In total, 137 (92%) of the referrals evaluated were from doctors, 3 (2%) came from nurses, 4 (3%) from psychologists, 1 (1%) from a social worker, and 4 (3%) from others (physiotherapists and dieticians). For referrals received from doctors, 84 (61%) of these practitioners were from the general medical department, followed by 32 (23%) practitioners from the general surgical department. The rest of the departments were largely similar in numbers. The full breakdown of the departments from which doctors referred their patients can be found in Table 2.

### 3.2. Patient

Patients who were referred during the evaluation were analysed for demographic and psychiatric information. These results are displayed in Table 3.

### 3.3. Outcomes

**Figure 1:**
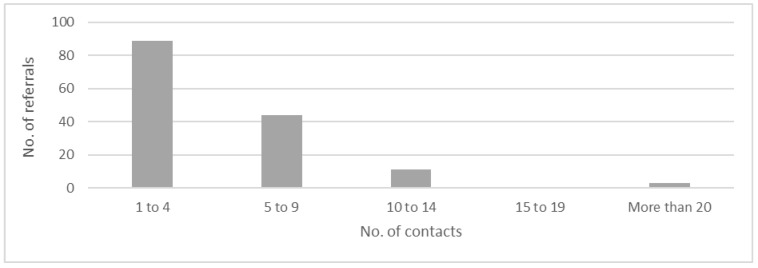
Number of contacts with CLP team for admission (total = 147).

The review outcomes of these referrals can be seen in Table 4, with a single referral often resulting in one or more of these review outcomes. In total, 89 (60%) of the referrals had 1–4 contacts, 44 (29%) had 5–9 contacts, 11 (7%) had 10–14 contacts, none had 15–19 contacts, and only 3 (2%) had more than 20 contacts with the CLP team (Figure 1). The mean (+/− standard deviation) length of hospitalisation of all 147 referrals was 31.5 (40.4) days. Five referrals were deceased during their hospitalisation and were not included in the calculations for the length of hospitalisations. The most frequent discharge destinations for these referrals were their own residences at 89 (60%), followed by residential aged care facilities at 32 (21%), with other discharge destinations including inter-hospital transfers, prison, and various forms of temporary accommodation. Follow-up mental health services were arranged for 51 (34%) of referrals, while the other 96 (66%) did not require any forms of follow-up with other mental health services. Of the 147 evaluated referrals, 139 (94%) of them were seen by the CLP team in under 24 h, 6 (4%) of them were seen within the 24-to-48 h period, while 2 (2%) referrals were seen past the 48 h mark.

## 4. Discussion

To our knowledge, this is the first study to assess the demands on a regional Australian CLP service to assist in the development of CLP standards. This study demonstrated the importance of the CLP service in addressing the psychiatric comorbidities and psychiatric challenges faced in the general hospital system. However, the service will require future resource influx to cope with the ageing population and increased referral numbers.

The referrers of the service are typically doctors, and the majority are from the general medical ward, as has previously been reported [20,21,22]. A recent systematic review found that poor recognition of mental illness was the most frequent reason for clinicians not referring to CLP [23]. We found that diagnostic clarification was the most common reason for referral to CLP, and this may be, again, due to minimal experience in dealing with psychiatric conditions or the presence of comorbid complex psychiatric conditions that are often best seen by psychiatrists [20]. This indicates a plausible deficiency in the ‘liaison’ aspects of the local CLP service that would require reform specifically in the areas of providing education in recognising common comorbid mental health conditions in general hospital inpatients.

Any healthcare service has an obligation to do no harm to patients. Knowing if a patient is on an MHA is a key factor, as it often greatly changes the management of their psychiatric conditions, e.g., removing their autonomy and making treatments compulsory [24]. Understanding a patient’s cultural background and beliefs are equally as important, as this improves the cultural safety of the mental health care delivered [25]. The rurality of a patient’s residence should also be considered, as mental health care provision for patients from rural and remote Australia differs from metropolitan centres [3]. In our study, a sizable proportion of patients requiring the CLP service were from rural locations, and this introduced additional complexity to their management. Resourcing and further collaborative care between CLP and community mental health services in the future could be an effective strategy for the delivery of safe mental health care to patients from rural and remote origins.

CLP service efficiency has previously been measured by the time taken to see the patient, with 95% of patients seen within 36 h in a Geneva hospital, 90% of referrals seen within 24 h at the Royal Melbourne Hospital, and a Perth metropolitan hospital, which stipulated a clinical indicator of 90% of referrals to be seen within 24 h and 95% of referrals within 36 h [26,27,28]. In our study, 94% of referrals were assessed within a 24 h timeframe by the CLP service. These referrals included some urgent mental health assessments seen within an hour. Referrals that were assessed in greater than 24 h may have been due to weekend or public holiday periods, as the local CLP team only operates on weekdays, or due to a lack of available CLP team members. The ability to maintain the CLP service’s efficiency will be challenging as the number of referrals is anticipated to rise without an appropriate increase in CLP resource allocations. CLP service is a referral-dependent service, and, hence, the time to respond to referrals is key. A positive relationship between timeliness of contact and LOS does exist in the domain of CLP, in that the LOS is significantly reduced with a timely referral to the CLP service, for both older and younger patients [2,12,29,30].

The age demographic with the highest referral rates were patients 60–79 years, and this showcases the challenges of an ageing population. The rapid trajectory of population ageing may negatively impact CLP service provision in the future, as elderly patients have been associated with extensive LOS, which could translate into increased CLP contacts and, thus, CLP resource usage [8,29]. In contrast, a study conducted internationally, which analysed a rapid assessment model of practice in CLP, showed that LOS was reduced in geriatric populations; however, this could have been due to the presence of a well-staffed and well-trained team [10]. International studies, specifically those in the United Kingdom, have demonstrated gaps in the provision of service, which later translated into positive policy and funding changes [17]. Meanwhile, a similar process has not yet been undertaken in Australia. Metropolitan services are often the first in line to benefit from structural and resource siphoning when it happens, which can obscure the dire needs of the CLP services in regional counterparts facing continued population growth and ageing populations.

Increased rurality is associated with an increase in the lack of staffing, leading to suboptimal service provision [17,30]. The importance of mental health care in the general hospital system is receiving greater recognition from healthcare staff and key stakeholders, and the modification of standards such as the National Safety and Quality Health Service Standards reflect this [12,17]. However, with the ongoing trajectory of the rise in population numbers and, specifically, the ageing population numbers, regional and rural CLP services may find it increasingly difficult to achieve these standards, which could pose a difficulty in maintaining accreditation for the training of psychiatric registrars, which are paramount in regional psychiatric service delivery [8]. Additionally, the Independent Hospital Pricing Authority recently found that comorbid mental health conditions are not currently associated with increased payments to the hospital due to the poor identification of inpatients [17]. Hence, with the already evident and anticipated increased use of the CLP service, an enhancement of the service would lead to positive changes in mental health care recognition and payment schemes for hospitals, a symbiosis between CLP and hospital administrators.

This study is not without its limitations. The liaison aspect was not officially evaluated in this study, as this could be examined through a subjective analysis from a consumer’s perspective, and this will be evaluated in a follow-up study through the use of a referrer satisfaction survey. Hence, this evaluation alone should not be representative of the CLP’s liaison aspect that it currently provides. Our study was subject to limitations related to the retrospective study design, particularly the descriptive nature of this study, which meant we could not quantify the degree of benefit the CLP service provided to patients. In addressing this, a prospective study tracking the outcomes of patients following CLP intervention is recommended to expound the findings of the current study. Additionally, this study provides information on referrer and patient characteristics from a single service, and the findings from this study may not be representative of other services. However, the tool that has been developed for this study will be of great value in future larger studies. Furthermore, without a standardised Australian model for CLP service evaluation, this would pose a barrier to accurately benchmarking services and identifying key knowledge gaps.

## 5. Conclusions

In conclusion, our study described and evaluated a regional Australian CLP service. The local CLP team currently operates with limited resource provision, and the sustainability of the service will become challenging as the population continues to age without an appropriate influx of resources. This study also emphasises the importance of conducting a broad-based evaluation of CLP services. We believe our study findings will contribute to future studies by providing a comprehensive method for evaluating other CLP services, with the goal of developing nationally accepted CLP benchmarks that are currently lacking.

## Figures and Tables

**Table 1:** Service evaluation tool.

**Referral**	
1. Referrer	Doctor Nurse Psychologist Social Worker Other
2. Referrer Department	Medical Surgical Orthopaedic Oncology Obstetrics and Gynaecology Intensive Care Unit Coronary Care Unit
3. Medical reason for admission under treating team	
4. Reason for referral to CLP Team (Select all those that apply)	Diagnostic Clarification/Review Medication Review (Cease, altering dose, addition of medications) Risk Assessment
5. Time taken for CLP team to review patient	<24 h 24–48 h >48 h
**Patient**	
6. Gender	Male Female Other
7. Age of patient	18–39 years 40–59 years 60–79 years 80–100 years
8. Indigenous Status of patient	Non-indigenous Aboriginal Torres Strait Islander South Sea Islander
9. Mental Health Act (MHA) status	Voluntary or None Recommendation for Treatment Emergency Examination Authority Treatment Authority
10. Transfer from rural origin	Yes No
11. Previous psychiatry contact	Yes No
12. Established existing psychiatry diagnosis	Yes No
**Outcome**	
13. Review outcome (Select all those that apply)	Diagnosis and category; (Mood disorders, psychotic disorders, cognitive disorders, other psychiatric conditions) Non-pharmacological management advice Pharmacological management advice (Cease, altering dose, addition of medications) Admission to Mental Health Unit Referral to other services (Psychologist, other allied health professions)
14. Number of contacts with CLP this admission	1–4 5–9 10–14 15–19 ≥20
15. Total length of hospitalization (Days)	
16. Discharge destination	Home Residential Aged Care Facility Hospital Transfer Others
17. Follow-up contact with mental health services	Yes No

**Table 2:** Departments submitting CLP referrals.

Hospital Departments	Number of Referrals (%)
General Medical	84 (61%)
General Surgical	32 (23%)
Orthopaedics	5 (4%)
Coronary Care Unit	7 (5%)
Obstetrics and Gynaecology	2 (1%)
Oncology	2 (1%)
Intensive Care Unit	5 (4%)
Total	137

**Table 3:** Patient characteristics.

Gender	Number of Referrals
Male	75 (51%)
Female	72 (49%)
**Age**	
18–39 years	23 (16%)
40–59 years	37 (25%)
60–79 years	57 (38%)
80–100 years	30 (21%)
**Indigenous status**	
Non-Indigenous	144 (98%)
Aboriginal	3 (2%)
Torres Strait Islander	0 (0%)
South Sea Islander	0 (0%)
**Mental health status**	
Voluntary or none	133 (91%)
Recommendation for assessment	8 (6%)
Emergency examination authority	4 (2%)
Treatment authority	2 (1%)
**Rural origin**	
Yes	31 (22%)
No	116 (78%)
**Previous psychiatry contact**	
Yes	71 (48%)
No	76 (52%)
**Established existing psychiatric diagnoses**	
Yes	87 (59%)
No	30 (41%)

**Table 4:** Review outcome (multiple outcomes may be selected for a single referral).

Review Outcome	Number of Referrals
Diagnosis and category - Mood disorder - Psychotic disorders - Cognitive disorders - Other psychiatric conditions	137 (91%) 36 (24%) 9 (6%) 63 (43%) 29 (20%)
Non-pharmacological management advice	33 (22%)
Pharmacological management advice	120 (81%)
Admission to mental health unit	8 (5%)
Referral to other services - Psychologist - Other allied health professions	77 (52%) 41 (28%) 36 (24%)

## Data Availability

The datasets analysed in this study are available from the corresponding author upon reasonable request.
